# Femoral radiographic indices for pre-operative osteoporosis screening in postmenopausal female patients undergoing total hip arthroplasty for osteoarthritis

**DOI:** 10.1371/journal.pone.0341658

**Published:** 2026-01-28

**Authors:** Masanori Nishi, Yasushi Yoshikawa, Yuki Usui, Hajime Nishida, Shota Nakamura, Koichiro Tashiro, Yoshifumi Kudo

**Affiliations:** Department of Orthopaedic Surgery, Showa Medical University, Tokyo, Japan; Showa University, JAPAN

## Abstract

The purpose of this study was to investigate the correlation between femoral morphological indices from anteroposterior hip radiographs and dual-energy X-ray absorptiometry-assessed bone mineral density in postmenopausal female patients undergoing primary total hip arthroplasty for hip osteoarthritis. We also evaluated the impact of hip deformity on these correlations and the diagnostic cut-off values for osteoporosis. This retrospective study, conducted at a single institute (February 2018 to July 2024), reviewed the data of postmenopausal patients (>50 years old) with hip osteoarthritis who underwent total hip arthroplasty. Patients with a history of hip surgical procedures, infection, metabolic bone disease, or inadequate imaging findings were excluded. Dual-energy X-ray absorptiometry was used to assess bone mineral density at the femoral neck, total hip, lumbar spine, and distal radius. Five femoral indices were measured: the canal-to-calcar ratio, canal flare index, cortical thickness index, canal diaphysis ratio, and canal bone area ratio. Analyses included Pearson’s correlation and receiver operating characteristic curve analysis. Moderate correlations were observed between total hip bone mineral density and indices in 95 hip osteoarthritic joints (all Tönnis grade 3) and 86 normal joints. The canal bone area ratio had the strongest correlation (hip osteoarthritis: r = −0.61; normal: r = −0.62; p < 0.01). Receiver operating characteristic analysis produced area under the curve values of 0.75 (cutoff: 0.47; p < 0.01) for hip osteoarthritis and 0.74 (cutoff: 0.49; p < 0.01) for normal hips. Femoral indices, particularly the canal bone area ratio, cortical thickness index and canal diaphysis ratio, moderately correlated with total hip bone mineral density even in patients with hip osteoarthritis and served as simple and effective screening tools for osteoporosis when pre-operative dual-energy X-ray absorptiometry was unavailable.

## Introduction

Osteoporosis is more common in postmenopausal women, and the gold standard for bone mineral density (BMD) assessment is dual-energy X-ray absorptiometry (DXA), which evaluates the bone density of the hip and lumbar spine [1]. The prevalence of osteoporosis in patients undergoing total hip arthroplasty (THA) is reportedly between 8% to 33% [2–5]. Additionally, a significant number of patients remain untreated pre-operatively owing to the lack of BMD evaluation [4,5]. Recently, with the extension of the average life expectancy and advancements in surgical techniques, THA is increasingly performed in older patients who are likely to have osteoporosis [6]. Osteoporosis in patients undergoing THA may influence the incidence of intraoperative periprosthetic fractures, stem subsidence, aseptic loosening, revision rates, and patient satisfaction with the procedure [4,7]. Therefore, it is crucial for surgeons to evaluate BMD pre-operatively as the osteoporotic condition influences the choice of implants, which contributes to the successful operative outcome. However, performing pre-operative BMD assessments for all patients undergoing THA may be challenging owing to cost considerations, and access to such evaluations may be limited in developing countries [8].

Different femoral morphological indices using anteroposterior (AP) radiographs of the proximal femur have been proposed to assess femoral bone density. The Singh index and Dorr classification are well-established and widely used indices [9,10]. Other indices such as the cortical thickness index (CTI), canal flair index (CFI), and canal-to-calcar ratio (CCR) have also been reported [8,11–13].

Bone sclerosis and osteophyte formation in patients with hip osteoarthritis (HOA) can make measuring BMD difficult [14,15]. Although some studies have investigated the relationship between pre-operative BMD and femoral indices in patients with HOA, no detailed examination of the relationship with DXA measurement sites or of the difference between patients with and without degenerative changes has been performed [8]. Thus, we hypothesized that even in patients with HOA, femoral morphological indices could be utilized for osteoporosis screening. We investigated whether the correlation between BMD at each measurement site and femoral indices on AP hip radiographs had an impact on degenerative changes in the hip joints. Furthermore, we pre-operatively identified patients with osteoporosis to establish suitable cutoff values for these measurements using receiver operating characteristic (ROC) curve analyses. The overall aim of this work was to investigate correlations between femoral morphological indices from AP hip radiographs and DEXA-assessed BMD in postmenopausal female patients undergoing primary THA for HOA.

## Materials and methods

### Patients

This retrospective study was approved by the Ethics Committee of Showa Medical University (No 2024–278-B) and has been performed in accordance with the ethical standards laid down in the 1964 Declaration of Helsinki. Informed consent was obtained using an opt-out method approved by the Ethics Committee of Showa Medical University (No 2024–278-B). Study details were made publicly available via the hospital website or public notice, and participants had the opportunity to decline participation. Written or verbal individual consent was not obtained, as permitted by the ethics committee. The medical records were accessed in March 2025 for research purposes. The authors had access to identifiable information during data collection, but all data were anonymized prior to analysis. This study included patients who underwent primary THA for HOA at Showa Medical University Hospital between February 2018 and July 2024. We diagnosed HOA based on radiographic evidence (progressive articular cartilage degradation, osteophyte formation, subchondral sclerosis, and joint space narrowing) and clinical manifestations (hip discomfort and limited range of motion). The exclusion criteria were male patients; patients aged <50 years; patients presenting HOA after fractures (femur, acetabulum, and pelvic ring); patients who underwent previous hip operations; patients who developed post-surgical infection, rapidly destructive coxarthrosis, or subchondral insufficiency fracture of the femoral head; patients with metabolic bone diseases; patients who did not have an appropriate AP radiograph of the hip joint; and patients who did not undergo DXA within three months prior to the operation.

### BMD measurement

At our institution, all patients scheduled for THA routinely undergo DXA for bone strength assessment, except in cases in which pre-operative DXA cannot be performed because of the rapid deterioration of symptoms. DXA was performed using Lunar Prodigy (GE Healthcare, Madison, WI, USA) or Hologic QDR Discovery (Hologic, Waltham, MA, USA). Among the total study population, 130 cases were measured using the Hologic QDR Discovery device. Conversion equations published by Xu et al. [16] were applied to harmonize BMD values across devices. Separate regression models were used for lumbar spine, femoral neck (FN), and total hip (TH) regions to convert measurements from one device to another. Subsequent calculation of T-scores was based on device-specific reference means and standard deviations, using a Japanese reference database [17]. Our institute’s DXA measurement protocol involves imaging the left femur. For this study, if the left side already presented an implant, the right femur was imaged. Cases in which DXA was performed on the non-surgical side with existing deformities or post-osteotomy conditions were excluded. DXA was performed using the femur measured at the TH and FN, the lumbar spine (as the average at L2-4), and the distal third of the radius. Based on the World Health Organization criteria, osteoporosis was defined as a T-score of ≤ −2.5, osteopenia as −2.5 < T-score <−1.0, and normal as ≥ −1.0, using measurements from either the lumbar spine or proximal femur. All evaluations were conducted within 6 weeks before the surgical procedure.

### Radiograph measurement

**Fig 1:**
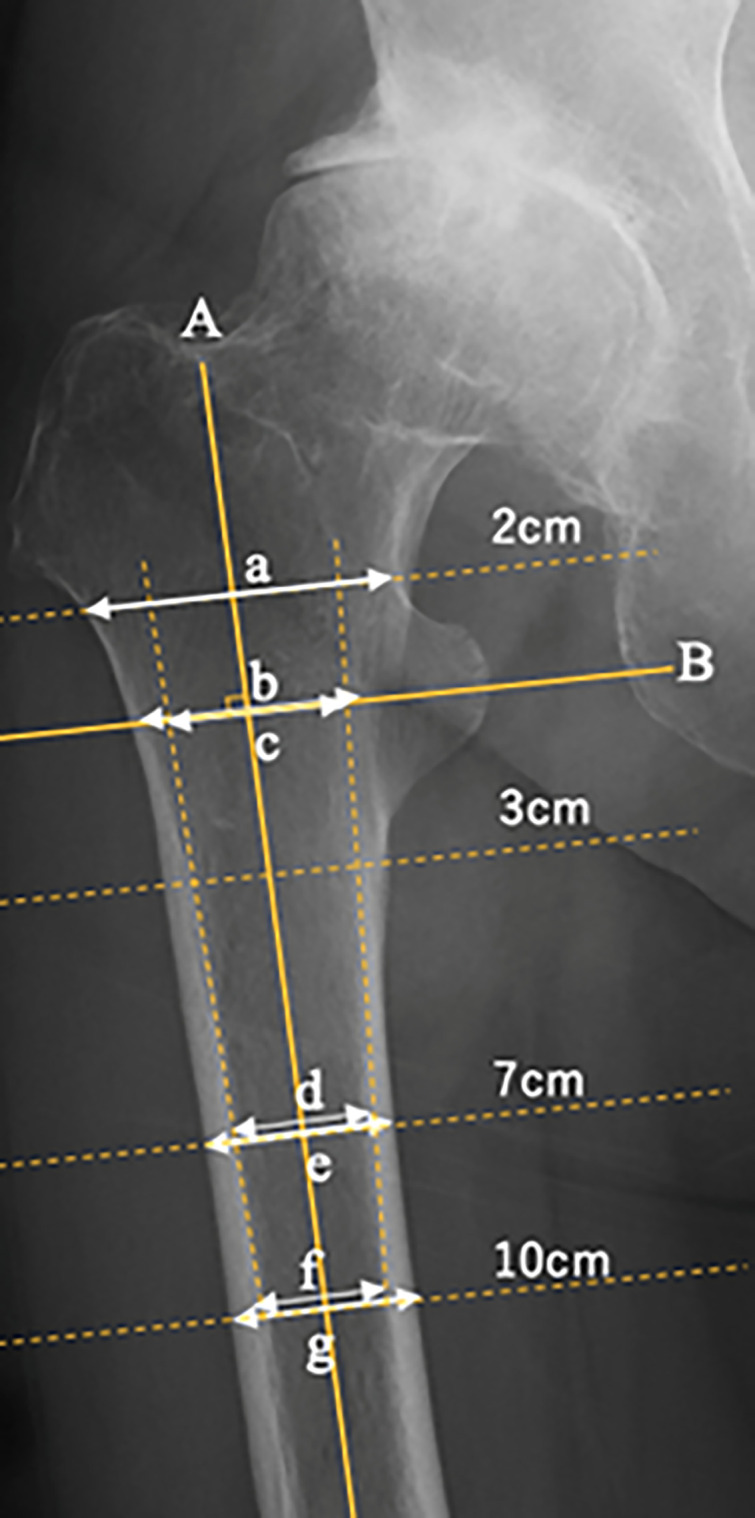
Proximal femoral morphological indices. Radiographic femoral indices included canal-to-calcar ratio (CCR): f/b; canal diaphysis ratio (CDR): d/e; canal flair index (CFI): a/f; cortical thickness index (CTI): f/g; canal bone area ratio (CBAR): (d + f)/ (e + g); Line A: femoral axis; Line B: perpendicular to femoral axis at lesser trochanter midpoint.

AP hip radiographs were obtained with the patients in the supine position and the lower limbs (hips) rotated internally by 15°. All radiographic images were acquired within 6 weeks prior to surgery. Proximal femoral morphological indices were evaluated using DICOM for the following parameters: CCR, CFI, CTI, canal diaphysis ratio (CDR), and canal bone area ratio (CBAR) (Fig 1). These parameters were measured on the side in which the BMD was assessed. The measurements were performed by an orthopedic surgeon. Previous studies have shown good intra- and interobserver reliabilities of the femoral morphological indices [8,12,18].

### Statistical analysis

Statistical analyses were performed using the JMP Pro v.17 for Mac (SAS Institute, USA). Patient characteristics are described using frequencies and percentages for categorical variables, and means with standard deviations, or medians for continuous variables, as appropriate. Normality was confirmed using the Shapiro-Wilk test. Correlations between the femoral indices and T-scores were assessed using Pearson’s correlation coefficients as follows: ± 1.0–0.9: very high, ± 0.9–0.7: high, ± 0.7–0.5: moderate, ± 0.5–0.3: low, ± 0.3–0.0: negligible correlation [18]. ROC curve analysis was used to evaluate the diagnostic value of the femoral indices for osteoporosis, and the area under the curve (AUC) was calculated to assess reliability. The cut-off values were determined when the Youden index was at its maximum [19]. Statistical significance was defined as p < 0.05.

## Results

**Fig 2:**
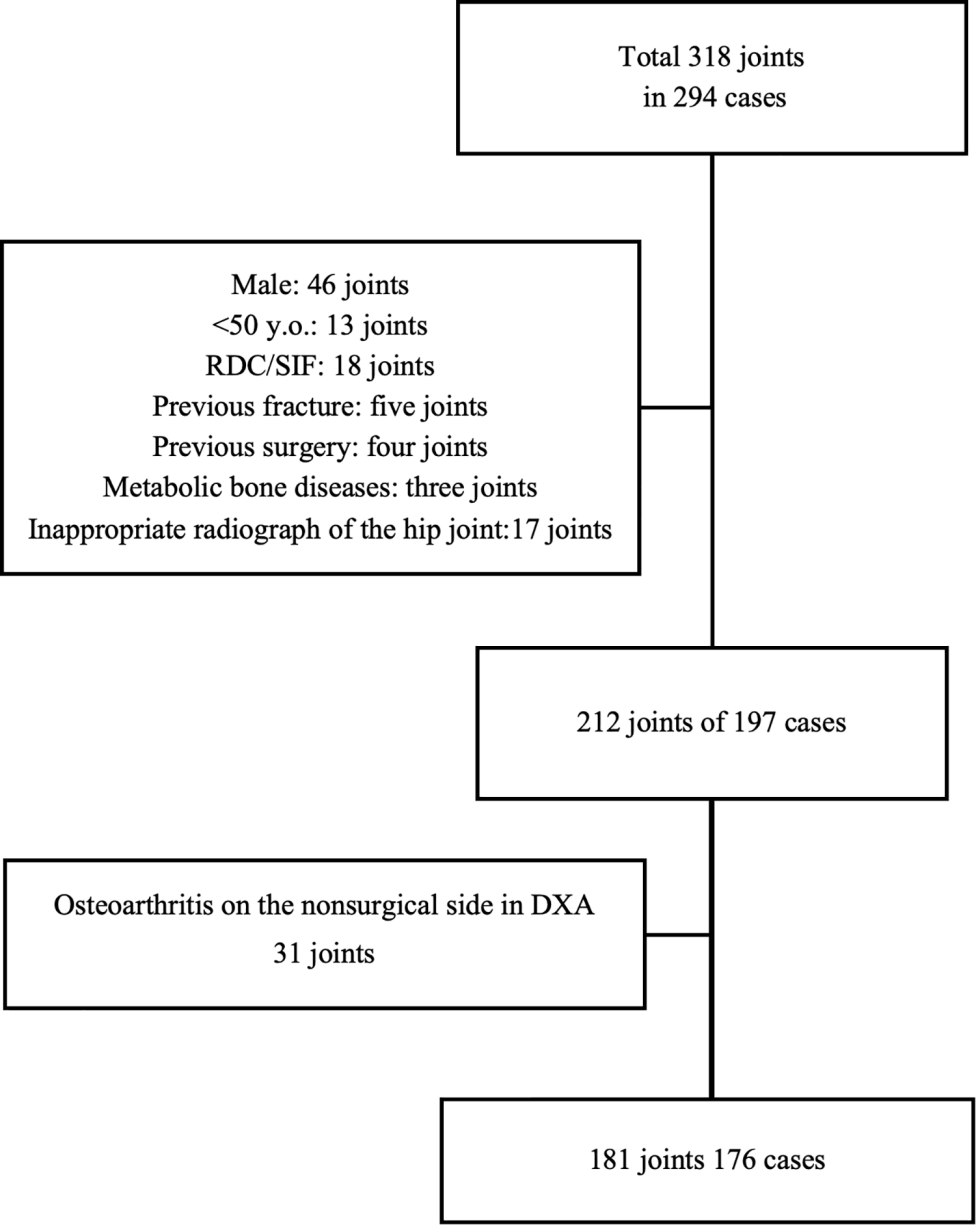
Patient selection flowchart. RDC, rapidly destructive coxarthropathy; SIF; subchondral insufficiency fracture; DXA, dual-energy X-ray absorptiometry.

**Fig 3:**
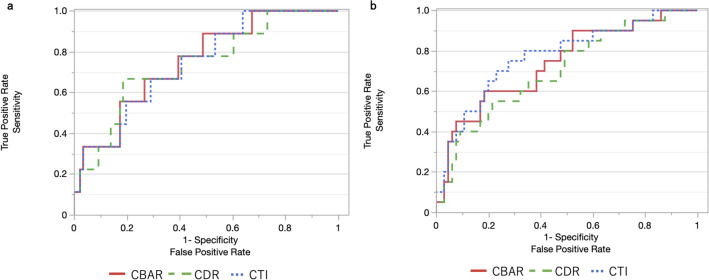
Receiver operating characteristic (ROC) curve of femoral indices (CBAR, CDR, CTI) for osteoporosis diagnosis. (a) surgical side, (b) non-surgical side. CBAR, canal bone area ratio; CDR, canal diaphysis ratio; CTI, cortical thickness index.

The patient selection process and background information are shown in Fig 2 and Table 1. A total of 181 joints in 176 cases were analyzed. BMD of the hip was assessed in 95 joints of 90 cases on the surgical side, and all patients were diagnosed with Tönnis grade 3 HOA; 86 joints of 86 cases on the non-surgical side, where osteoarthritis changes were not observed, were also assessed (Fig 2) [20]. The T-scores for each measurement site and the results of the bone turnover markers are shown in Table 1. Results of each femoral morphological indices and the correlation between the T-scores at each measurement site and the femoral indices are shown in Table 2. The highest correlation coefficient was observed between the TH and CBAR, indicating a moderate correlation (surgical side: r = −0.61, 95% confidence interval [CI]: −0.72, −0.46, p < 0.01; nonsurgical side: r = −0.62, 95% CI: −0.74, −0.47, p < 0.01). The results of the ROC analysis for osteoporosis diagnosis are depicted in Fig 3. The results of the femoral indices analysis including the AUC and cutoff values are shown in Table 3. All AUC values were ≥0.7, indicating that the femoral indices served as a good screening tool.

**Table 1:** Patient Demographics.

Number of joints (patients)	Total n = 181 (176)	Surgical site n = 95 (90)	Non-surgical site n = 86 (86)
Age [years] median (range)			
BMI [kg/m²] median (range)	23.7 (16.8, 37.5)	24.0 (16.8, 34.0)	23.6 (16.8, 37.5)
Smoking, n (%)	11(6)	7 (8)	4 (4)
Diabetes, n (%)	26 (14)	14 (16)	12 (13)
T- scores, median (range)			
Total hip	−1.2 (−4.0, 2.9)	−1.4 (−3.3, 1.2)	−1.1 (−4.0, 2.9)
Femoral neck	−1.2 (−3.9, 4.6)	−1.9 (−3.9, 0.6)	−0.6 (−2.8, 4.6)
Lumbar	−0.6 (−3.9, 3.4)	−0.8 (−3.9, 3.4)	−0.6 (−3.2, 3.0)
Distal radius	−1.4 (−5.4, 3.2)	−1.9 (−5.4, 2.1)	−1.0 (−4.4, 3.2)
WHO criteria N(%)			
Osteoporosis	29 (16)	20 (23)	9 (9)
Osteopenia	102 (56)	55 (64)	47 (49)

BMI, body mass index; SD, standard deviation; WHO, World Health Organization.

**Table 2:** Results of each femoral morphological indices and Correlation Coefficients Between Bone Mineral Density Measurement Sites and Femoral Indices.

	CCR	CDR	CFI	CTI	CBAR
Non-surgical side median (range)	0.42 (0.30-0.60)	0.48 (0.34-0.58)	0.28 (0.19-0.39)	0.46 (0.31-0.59)	0.47 (0.32-0.57)
Surgical site median (range)	0.45 (0.31-0.69)	0.48 (0.31-0.76)	0.27 (0.15-0.44)	0.46 (0.30-0.77)	0.48 (0.31-0.76)
**Correlation Coefficients (r)**					
Total hip, median (95% CI) Non-surgical side	−0.30 (−0.48, −0.10) p < 0.01	−0.59 (−0.72, −0.44) p < 0.01	−0.45 (−0.61, −0.26) p < 0.01	−0.62 (−0.73, −0.47) p < 0.01	−0.62 (−0.74, −0.47) p < 0.01
Total hip, median (95% CI) Surgical site	−0.13 (−0.32, 0.08) p = 0.04	−0.61 (−0.72, −0.47) p < 0.01	−0.26 (−0.44, −0.06) p = 0.01	−0.57 (−0.69, −0.42) p < 0.01	−0.61 (−0.72, −0.46) p < 0.01
Femoral neck, median (95% CI) Non-surgical side	−0.29 (−0.47, −0.09) p < 0.01	−0.59 (−0.43, −0.24) p < 0.01	−0.37 (−0.54, −0.17) p < 0.01	−0.44 (−0.60, −0.25) p < 0.01	−0.45 (−0.60, −0.26) p < 0.01
Femoral neck, median (95% CI) Surgical site	−0.21 (−0.40, −0.01) p = 0.23	−0.35 (−0.51, −0.16) p < 0.01	−0.08 (−0.28, 0.12) p = 0.42	−0.31 (−0.48, −0.12) p < 0.01	−0.34 (−0.51, −0.15) p < 0.01
Lumber, median (95% CI)	−0.22 (−0.36, −0.07) p < 0.01	−0.40 (−0.51, −0.26) p < 0.01	−0.20 (−0.33, −0.05) p < 0.01	−0.38 (−0.50, −0.25) p < 0.01	−0.40 (−0.51, −0.27) p < 0.01
Distal radius, median (95% CI)	−0.29 (−0.42, −0.15) p < 0.01	−0.45 (−0.56, −0.32) p < 0.01	−0.30 (−0.43, −0.16) p < 0.01	−0.42 (−0.53, −0.29) p < 0.01	−0.44 (−0.55, −0.32) p < 0.01

CCR, canal-to-calcar ratio; CDR, canal diaphysis ratio; CFI, canal flap index; CTI, cortical thickness index; CBAR, canal bone area ratio; CI, confidence interval.

**Table 3:** Area Under the Curve and Cut-off Values of Femoral Indices (CBAR, CDR, CTI) on the Surgical and Non-surgical Sides.

	Femoral indices	AUC	95% CI LCL-UCL	sensitivity	specificity	Positive predictive value	Negative predictive value	Cut-off value	p value
Surgical side	CBAR	0.75	0.56-0.88	0.51	0.90	0.98	0.16	0.47	< 0.01
	CDR	0.74	0.53-0.88	0.81	0.68	0.96	0.67	0.53	< 0.01
	CTI	0.74	0.56-0.87	0.71	0.68	0.95	0.19	0.48	< 0.01
Non-surgical side	CBAR	0.74	0.59-0.85	0.82	0.60	0.87	0.50	0.49	< 0.01
	CDR	0.70	0.56-0.82	0.79	0.55	0.85	0.44	0.51	< 0.01
	CTI	0.77	0.63-0.87	0.73	0.75	0.91	0.45	0.47	< 0.01

AUC, area under the curve; CI, confidence interval; LCL-UCL, lower and upper bounds 95% confidence interval; CBAR, canal bone area ratio; CDR, canal diaphysis ratio; CTI, cortical thickness index.

## Discussion

In this study, proximal femoral morphological indices were identified as useful screening tools for osteoporosis in patients with HOA undergoing THA, particularly for postmenopausal females at a high risk for osteoporosis. We found moderate correlations between the femoral indices and T-scores at the TH site on both the arthritic and non-arthritic hip sides. These findings suggest that morphological parameters may serve as practical indicators of BMD, potentially enabling clinicians to identify patients with compromised BMD before performing THA. Importantly, our cohort consisted of patients with advanced HOA (Tönnis grade 3). Severe deformity often limits the applicability of conventional morphological evaluation; however, our results demonstrated that these femoral indices remained useful even in such advanced-stage HOA. This represents a meaningful clinical strength of the present study.

Previous research has revealed the association between femoral morphological parameters and osteoporosis in patients with HOA [8,13]. Sah et al. [21] investigated 32 Caucasian postmenopausal women and found a moderate correlation (r = −0.58) between the CTI and the femoral neck T-score. Additionally, Rohe et al. analyzed 216 Caucasian patients (107 men and 109 women) and reported that CBR-7 and CBR-10 showed strong correlations with the femoral neck T-score in both men (r = −0.60, r = −0.55, respectively) and women (r = −0.74, r = −0.77, respectively) [13]. Liu et al. [8] investigated 81 Asian patients (mean age 57.8 years, range: 29–82) and demonstrated a correlation (r = −0.63) between CBR-10 and the proximal femur T-score. The correlation coefficients in our study are generally consistent with those found in previous reports. Furthermore, the indices that showed relatively strong correlations were similar to those reported in previous studies, thus providing further evidence supporting the utility of CBAR, CDR, and CTI. These other reports did not consider the HOA grade; however, our results revealed that even in patients presenting advanced HOA (Tönnis grade 3), the femoral indices retained a moderate correlation with the T-scores, indicating that these measurements can capture key aspects of bone health despite degenerative changes in the hip joint.

Additionally, our results revealed that the TH site had a stronger correlation than the FN site. Glowacki et al. [14] compared bilateral hip BMD in patients with unilateral HOA and found that BMD at the FN site was predominantly higher on HOA side, whereas no significant bilateral differences were observed for the TH site. Their findings may influence and support the interpretation of the results of the present study. Accurate assessment of BMD can be challenging in patients with HOA owing to the influence of osteophytes and bone cysts. Our results also showed a slightly higher correlation coefficient in the non-arthritic hip. Although the non-arthritic hip side showed a better correlation coefficient than that of the surgical side, the difference was not significant. Based on our findings, in patients presenting bilateral hip involvement, morphological indices of the femur on the surgical side may be helpful, whereas in patients with unilateral involvement, using indices from the non-arthritic hip side may allow for more accurate screening for osteoporosis. However, correlations with the lumbar spine and radius were slightly inferior to those involving the femur. Limited research has been conducted examining the correlation between the femoral indices and T-scores at the distal radius [22]. Despite the lack of previous reports evaluating the radius, the BMD in the radius tends to vary more than that observed in the femur and lumbar spine, and its usefulness in THA patients remains unclear. However, reports have suggested that the radius may be useful as a screening site and that it may be associated with prosthetic hip subsidence, indicating the need for further research [3,23].

In addition to AUC values, we evaluated sensitivity, specificity, positive predictive value (PPV), and negative predictive value (NPV) to determine the overall clinical utility of each index. On the surgical side, CBAR and CTI showed very similar discriminatory performance for osteoporosis (AUC: 0.75 vs. 0.74), with a negligible difference of 0.01. Their validity metrics also showed complementary strengths: CBAR demonstrated a markedly higher specificity (0.90) and PPV (0.98), whereas CTI showed a higher sensitivity (0.71). On the non-surgical side, CTI showed the highest AUC (0.77), and its sensitivity and specificity (0.73 and 0.75) were well balanced compared with the other indices. These results indicate that no single index clearly outperformed the others, and the small differences in AUC among CBAR, CDR, and CTI did not translate into substantial differences in their overall validity profiles. Furthermore, because CTI requires measurement at only one level and is technically simpler than CBAR, its practicality may be advantageous in routine clinical settings.

Regarding other osteoporosis screening tools, several studies have demonstrated that the Osteoporosis Self-Assessment Tool for Asians (OSTA), a simple screening tool requiring only age and body weight, achieves good diagnostic performance for osteoporosis and is practical in various clinical settings [24]. In hip surgery populations, Higuchi et al. [25] reported that OSTA alone showed favorable discrimination (AUC: 0.817), and that combining OSTA with subjective visual assessment of hip radiographs by orthopedic surgeons further improved the AUC to 0.821–0.915. It should be noted that their cohort included patients with varying degrees of hip pathology and did not consist exclusively of individuals with advanced-stage HOA. In contrast, all patients in the present study had advanced HOA, where substantial changes in proximal femoral morphology may affect radiographic measurements. Similarly, Liu et al. [26] reported that combining the Singh index with OSTA improved the AUC (0.795) compared with either method alone (OSTA 0.534; Singh 0.636). Further studies are warranted to determine the optimal combination of radiographic parameters and simple clinical tools in orthopedic practice.

Reports on BMD evaluation using artificial intelligence (AI) are becoming increasingly common [27]. The advantage of our method is that it is a simple screening tool that relies only on AP radiographs of the hip. However, with future technological advancements, screening will likely become simpler and more accurate even in patients with HOA. Although the use of AI may yield excellent results, such technology is not yet readily available in clinical practice. Our study aimed to perform a preliminary diagnostic screening of osteoporosis using simple tests based on the findings from plain radiographs, which are frequently used in daily practice and are widely available in healthcare facilities. We showed a concordance between hip T-scores and osteoporosis findings obtained from direct radiographs.

This study had some limitations. First, this was a retrospective study that included only postmenopausal women. In addition, our sample size was small. Further large-scale studies are required. Second, the study population was limited to East Asian individuals. Given that BMD is influenced by race, the results of this study may not apply to other ethnicities. Third, the effect of lower limb rotation during radiography cannot be completely excluded. While all radiographs were obtained using a standardized protocol of 15° internal hip rotation and conducted by trained radiologic technologists, minor variations in positioning may have occurred. As hip rotation has been shown to influence BMD measurements [28], the potential impact of inconsistent positioning cannot be fully disregarded. Utilizing dedicated positioning jigs and footrests could potentially minimize this source of variability in future studies [29]. The calibration equations used in this study were derived from Xu et al. [16], which involved BMD measurements from GE Lunar iDXA and Hologic Discovery devices. Systematic errors may remain when applying these equations to populations with different demographic characteristics or extreme bone density values. Additionally, our lumbar spine measurements included only vertebral levels L2–L4, whereas the original equations were based on L1–L4, which may affect the accuracy of the conversion. These factors should be considered as limitations when interpreting the converted BMD and corresponding T-scores. Finally, we did not measure the intra- and interobserver reliability. However, previous studies have reported intraclass correlation coefficients of approximately 0.9, indicating that these indices had minimal measurement errors [8,12].

Although DXA is undoubtedly the gold standard for assessing BMD, our findings suggest that when pre-operative measurements are challenging, AP hip radiographs, which are always obtained prior to THA, can be used for evaluation. Specifically, femoral indices (CBAR, CTI, and CDR) may be useful screening tools for osteoporosis.

## Conclusion

In this study, we hypothesize that proximal femoral morphological indices could be used to screen for osteoporosis in patients with HOA undergoing THA, particularly in postmenopausal females at high risk for osteoporosis. Our findings support this hypothesis, as demonstrated by the moderate correlations observed between the femoral indices and T-scores at the TH site on both the surgical and non-surgical sides. These results suggest that morphological parameters may serve as practical indicators of BMD, potentially enabling clinicians to identify patients with compromised BMD before THA is performed. Moderate correlations are observed between the BMD of the TH site and femoral indices, even in advanced-stage HOA. Thus, when pre-operative DXA measurements are difficult to obtain, femoral morphological indices (CBAR, CTI, and CDR) may be useful and simple screening tools.

## References

[1] Krueger D, Tanner SB, Szalat A, Malabanan A, Prout T, Lau A, et al. DXA Reporting Updates: 2023 Official Positions of the International Society for Clinical Densitometry. J Clin Densitom. 2024;27(1):101437. doi: 10.1016/j.jocd.2023.101437 38011777

[2] Karachalios TS, Koutalos AA, Komnos GA. Total hip arthroplasty in patients with osteoporosis. Hip Int. 2020;30(4):370–9. doi: 10.1177/1120700019883244 31672068

[3] Lingard EA, Mitchell SY, Francis RM, Rawlings D, Peaston R, Birrell FN, et al. The prevalence of osteoporosis in patients with severe hip and knee osteoarthritis awaiting joint arthroplasty. Age Ageing. 2010;39(2):234–9. doi: 10.1093/ageing/afp222 20032523

[4] Bernatz JT, Brooks AE, Squire MW, Illgen RI 2nd, Binkley NC, Anderson PA. Osteoporosis Is Common and Undertreated Prior to Total Joint Arthroplasty. J Arthroplasty. 2019;34(7):1347–53. doi: 10.1016/j.arth.2019.03.044 30992237

[5] Watanabe N, Miyatake K, Takada R, Ogawa T, Amano Y, Jinno T, et al. The prevalence and treatment of osteoporosis in patients undergoing total hip arthroplasty and the levels of biochemical markers of bone turnover. Bone Joint Res. 2022;11(12):873–80. doi: 10.1302/2046-3758.1112.BJR-2022-0252.R1 36464500 PMC9792872

[6] Ogino D, Kawaji H, Konttinen L, Lehto M, Rantanen P, Malmivaara A, et al. Total hip replacement in patients eighty years of age and older. J Bone Joint Surg Am. 2008;90(9):1884–90. doi: 10.2106/JBJS.G.00147 18762648

[7] Maier GS, Kolbow K, Lazovic D, Maus U. The Importance of Bone Mineral Density in Hip Arthroplasty: Results of a Survey Asking Orthopaedic Surgeons about Their Opinions and Attitudes Concerning Osteoporosis and Hip Arthroplasty. Adv Orthop. 2016;2016:8079354. doi: 10.1155/2016/8079354 27999686 PMC5141559

[8] Liu Y, Ma W-J, Huang K, Yang J, Zeng Y, Shen B. Radiographic indexes in AP hip radiographs prior to total hip arthroplasty reveal candidates with low BMD. Osteoporos Int. 2022;33(4):871–9. doi: 10.1007/s00198-021-06231-8 34775528

[9] Singh M, Nagrath AR, Maini PS. Changes in Trabecular Pattern of the Upper End of the Femur as an Index of Osteoporosis. The Journal of Bone & Joint Surgery. 1970;52(3):457–67. doi: 10.2106/00004623-197052030-00005 5425640

[10] Dorr LD, Faugere MC, Mackel AM, Gruen TA, Bognar B, Malluche HH. Structural and cellular assessment of bone quality of proximal femur. Bone. 1993;14(3):231–42. doi: 10.1016/8756-3282(93)90146-2 8363862

[11] Ilyas G, Ipci FB. Evaluation of the Relationship between Osteoporosis Parameters in Plain Hip Radiography and DXA Results in 156 Patients at a Single Center in Turkey. Diagnostics (Basel). 2023;13(15):2519. doi: 10.3390/diagnostics13152519 37568882 PMC10417530

[12] Faundez J, Carmona M, Klaber I, Zamora T, Botello E, Schweitzer D. Radiographic Assessment of Bone Quality Using 4 Radiographic Indexes: Canal Diaphysis Ratio Is Superior. J Arthroplasty. 2024;39(2):427–32. doi: 10.1016/j.arth.2023.08.037 37597819

[13] Rohe S, Böhle S, Matziolis G, Jacob B, Brodt S. Plain radiographic indices are reliable indicators for quantitative bone mineral density in male and female patients before total hip arthroplasty. Sci Rep. 2023;13(1):19886. doi: 10.1038/s41598-023-47247-w 37963967 PMC10645725

[14] Glowacki J, Tuteja M, Hurwitz S, Thornhill TS, LeBoff MS. Discordance in femoral neck bone density in subjects with unilateral hip osteoarthritis. J Clin Densitom. 2010;13(1):24–8. doi: 10.1016/j.jocd.2009.09.007 20171566

[15] Martini F, Lebherz C, Mayer F, Leichtle U, Kremling E, Sell S. Precision of the measurements of periprosthetic bone mineral density in hips with a custom-made femoral stem. J Bone Joint Surg Br. 2000;82(7):1065–71. doi: 10.1302/0301-620x.82b7.9791 11041603

[16] Xu W, Chafi H, Guo B, Heymsfield SB, Murray KB, Zheng J, et al. Quantitative Comparison of 2 Dual-Energy X-ray Absorptiometry Systems in Assessing Body Composition and Bone Mineral Measurements. J Clin Densitom. 2016;19(3):298–304. doi: 10.1016/j.jocd.2015.06.002 26206525

[17] Soen S, Fukunaga M, Sugimoto T, Sone T, Fujiwara S, Endo N, et al. Diagnostic criteria for primary osteoporosis: year 2012 revision. J Bone Miner Metab. 2013;31(3):247–57. doi: 10.1007/s00774-013-0447-8 23553500

[18] Mukaka MM. Statistics corner: A guide to appropriate use of correlation coefficient in medical research. Malawi Med J. 2012;24(3):69–71. 23638278 PMC3576830

[19] Youden WJ. Index for rating diagnostic tests. Cancer. 1950;3(1):32–5. doi: 10.1002/1097-0142(1950)3:1<32::aid-cncr2820030106>3.0.co;2-3 15405679

[20] Kovalenko B, Bremjit P, Fernando N. Classifications in Brief: Tönnis Classification of Hip Osteoarthritis. Clin Orthop Relat Res. 2018;476(8):1680–4. doi: 10.1097/01.blo.0000534679.75870.5f 30020152 PMC6259761

[21] Sah AP, Thornhill TS, LeBoff MS, Glowacki J. Correlation of plain radiographic indices of the hip with quantitative bone mineral density. Osteoporos Int. 2007;18(8):1119–26. doi: 10.1007/s00198-007-0348-6 17340218 PMC2778043

[22] Yeung Y, Chiu KY, Yau WP, Tang WM, Cheung WY, Ng TP. Assessment of the proximal femoral morphology using plain radiograph-can it predict the bone quality? J Arthroplasty. 2006;21(4):508–13. doi: 10.1016/j.arth.2005.04.037 16781402

[23] Nazari-Farsani S, Vuopio ME, Aro HT. Bone Mineral Density and Cortical-Bone Thickness of the Distal Radius Predict Femoral Stem Subsidence in Postmenopausal Women. J Arthroplasty. 2020;35(7):1877-1884.e1. doi: 10.1016/j.arth.2020.02.062 32205004

[24] Koh LK, Sedrine WB, Torralba TP, Kung A, Fujiwara S, Chan SP, et al. A simple tool to identify asian women at increased risk of osteoporosis. Osteoporos Int. 2001;12(8):699–705. doi: 10.1007/s001980170070 11580084

[25] Higuchi R, Uemura K, Kono S, Mae H, Takashima K, Abe H, et al. Osteoporosis screening using X-ray assessment and osteoporosis self-assessment tool for Asians in hip surgery patients. J Bone Miner Metab. 2025;43(2):158–65. doi: 10.1007/s00774-024-01569-5 39656248 PMC11993500

[26] Liu Z, Gao H, Bai X, Zhao L, Li Y, Wang B. Evaluation of Singh Index and Osteoporosis Self-Assessment Tool for Asians as risk assessment tools of hip fracture in patients with type 2 diabetes mellitus. J Orthop Surg Res. 2017;12(1):37. doi: 10.1186/s13018-017-0539-6 28253896 PMC5335822

[27] Jang R, Choi JH, Kim N, Chang JS, Yoon PW, Kim C-H. Prediction of osteoporosis from simple hip radiography using deep learning algorithm. Sci Rep. 2021;11(1):19997. doi: 10.1038/s41598-021-99549-6 34620976 PMC8497544

[28] Lekamwasam S, Lenora RSJ. Effect of leg rotation on hip bone mineral density measurements. J Clin Densitom. 2003;6(4):331–6. doi: 10.1385/jcd:6:4:331 14716045

[29] Goh JC, Low SL, Bose K. Effect of femoral rotation on bone mineral density measurements with dual energy X-ray absorptiometry. Calcif Tissue Int. 1995;57(5):340–3. doi: 10.1007/BF00302069 8564796

